# Computing and visually analyzing mutual information in molecular co-evolution

**DOI:** 10.1186/1471-2105-11-330

**Published:** 2010-06-17

**Authors:** Sebastian Bremm, Tobias Schreck, Patrick Boba, Stephanie Held, Kay Hamacher

**Affiliations:** 1 Interactive Graphics Systems, Dept. of Computer Science, Technische Universität Darmstadt, Germany; 2 Bioinformatics & Theo. Biology, Dept. of Biology, Technische Universität Darmstadt, Germany

## Abstract

**Background:**

Selective pressure in molecular evolution leads to uneven distributions of amino acids and nucleotides. In fact one observes correlations among such constituents due to a large number of biophysical mechanisms (folding properties, electrostatics, ...). To quantify these correlations the mutual information -after proper normalization - has proven most effective. The challenge is to navigate the large amount of data, which in a study for a typical protein cannot simply be plotted.

**Results:**

To visually analyze mutual information we developed a matrix visualization tool that allows different views on the mutual information matrix: filtering, sorting, and weighting are among them. The user can interactively navigate a huge matrix in real-time and search e.g., for patterns and unusual high or low values. A computation of the mutual information matrix for a sequence alignment in FASTA-format is possible. The respective stand-alone program computes in addition proper normalizations for a null model of neutral evolution and maps the mutual information to *Z*-scores with respect to the null model.

**Conclusions:**

The new tool allows to compute and visually analyze sequence data for possible co-evolutionary signals. The tool has already been successfully employed in evolutionary studies on HIV1 protease and acetylcholinesterase. The functionality of the tool was defined by users using the tool in real-world research. The software can also be used for visual analysis of other matrix-like data, such as information obtained by DNA microarray experiments. The package is platform-independently implemented in Java and free for academic use under a GPL license.

## Background

### Background of the analysis problem

The understanding of molecular evolution requires a detailed understanding of the dynamics *among *the constituents of a molecule during its evolution [1]. Computational biologists seek evolutionary signals in data sets of sequences of biomolecules and signatures of a correlated dynamic of the evolutionary processes shaping the characteristics of molecules under investigation. Such co-evolution occurs mostly, when either an amino acid or a nucleotide within a biomolecule evolves in concert with another "site" within the same or a partnering molecule. The mutual information (MI) is widely used to detect such correlated evolutionary dynamics. The computation of the MI (see section Information-theoretical measure for details) itself is straightforward. However, proper normalization needs to be carefully taken into account [2-6] for typical, finite-sized data sets. This issue is discussed in more detail below. The problem of analyzing the obtained mutual information values was, however, not tackled until now: as the MI is a quantification between any two sites within a protein, of e.g., N amino acids, for such a molecule we need to compute and to analyze  MI values. Even for modest sized proteins with *N *~ 100 this means to visualize 5050 real values. Typically one cannot easily grasp structures, scales, etc. in such large amounts of data.

### Visual-Interactive Approach and Matrix Visualization

Recently, visualization has been widely recognized as a promising approach to help analysts and researchers to better understand such large amounts of complex data. The approach suggests to have visual-interactive displays appropriately encode information using visual mappings; and let the user interactively manipulate these displays to navigate, drill-down, and explore [7-9].

According to the structure of the data to be visualized, different visual mappings are appropriate. Matrix visualization [10] is appropriate for large amounts of data elements for which pairwise relationships with quantitative attributes are given. By representing the quantitative value of each relationship by color, matrix visualization is highly scalable, ultimately representing each relationship by a single pixel.

Important problems to address in designing effective matrix visualization systems involve choosing an appropriate color scale [11], data preprocessing steps, and applying suitable sorting on the matrices to be visualized. The latter is specifically important, as is allows to make assessments on the overall structure of the relationships. Matrix sorting usually arranges rows and columns of the matrix by similarity, with an appropriate similarity function defined on the row and column vectors of the matrix.

Matrix visualization as a technique is well-known and to date has found its way into software systems such as R[12] or Matlab[13]. However, many implementations are focused on producing static images, offering only limited support for interactive parameter change and navigation in the matrix display by the user. We therefore developed a fully interactive matrix visualization system in Java. It allows the user to change important parameters and navigate the data on the fly by means of a two-stage zooming mechanism. Furthermore, we support the joint visualization of two matrices, supporting our specific analysis problem.

## Implementation

### Visualization

The implemented application generically supports two data matrices: one data and one weight matrix (see section Normalization & Weighting) which can be inspected individually or jointly. The general approach is to map the normalized matrix values to an appropriate color map and display it as a grid. While using color is typically not the first choice for representing absolute values, it allows comparative analysis of value ranges and provides a highly compact view of the overall data distribution. Specifically in the case of large data matrices and in conjunction with an appropriate matrix sorting mechanism it allows the assessment of the features of the matrix. The application supports detailed visualization of either one of the two matrices. Detailed information is available by interactively *zooming *into parts of the matrix and *restricting *the displayed data to specific value ranges. Thereby, the approach follows Shneiderman's Information Visualization Mantra ("Overview first - zoom, filter, refine - details on demand" [14]). Sorting the matrix by arranging rows and columns by similarity reduces its complexity and allows identification of systematic (similar) relationships between entities of the experiment by homogeneous colors [10].

Our sorting algorithm works by finding a so-called *seed *row according to the maximum of the sum of contained values. This row is made the top row. Then, the sorting algorithm among the remaining rows finds the one that is most similar to the seed row, where the degree of (dis)similarity is measured by the *l*_1 _norm between the respective row vectors. The algorithm places the found row just below the seed row, makes the found row the new seed row, and iteratively continues until all rows have been processed. The same approach is then applied on the columns of the matrix. This sorting algorithm is rather simple, yet provides a useful starting point for the visual analysis. The algorithm has quadratic runtime complexity. For an overview of the application design, please see the system illustration and description provided in section Results and Discussion.

### Mutual information computation

The stand-alone program micato (mutual information calculation tool) reads a sequence file in FASTA format and calculates the MI of the sequence contained therein. By separation from the visualization tool micato can be run on e.g., clusters using job-scheduling systems. This is useful in particular for sampling large instances of null models for normalization.

To this end micato calculates in a first step the sequence entropies of each column of the sequence alignment and stores it. Then the joint entropy of each pairing of two columns is calculated and by equation 1 the MI is calculated and stored in a matrix MI_*ij *_for a pair of positions (*i*, *j*). Then micato runs a user defined number of independent column shuffles to generate a statistically significant number of instances of the null model (see section Normalization & Weighting for details). The MI matrix is exported as a CSV file, as well as the *Z*-scores of those MI values with respect to the statistics of the null model. The CSV format can be read by the MIMatrixViz program without further conversion.

## Methods

### Information-theoretical measure

To measure co-evolution among residues one frequently uses the mutual information (MI), defined as [15]:(1)

where *x *and *y *are outcomes for random variables *X*_*i *_and *Y*_*j *_drawn from a symbol set , taken from a multiple sequence alignment as columns *i *and *j*. The symbols *H*_*i*_(*x*), *H*_*j *_(*y*) are the column sequence entropies and *H*_*ij *_(*x*, *y*) is the entropy computed by the joint probability function *p*_*ij*_(*x*, *y*). Repeated application of the equation leads to a symmetric MI matrix (MI_*ij*_) for all pairings (*i*, *j*).

In studies on proteins the symbol set consists of the 20 standard amino acids , which has to be expanded to include the gap character and an extra character for non-standard amino acids . We set the probabilities *p*_*i*_(*x*),*p*_*j*_(*y*), and *p*_*ij*_(*x*, *y*) to the observed frequencies of amino acids within the columns of a multiple sequence alignment. This can be done with the supplied routines in the program micato.

### Normalization & Weighting

Although in the post-genomic era [16] we have access to huge databases of sequences, in a typical setting the number of sequences available is still only of the order 10^2^. We have previously shown that this limitation might lead to substantial finite-size effects in the computed MI values [2]. These effects can be compensated by normalizing the obtained MI values to a null model of evolution [17].

We have implemented such a normalization by computing the MI for *shuffled *columns: while maintaining the one-column sequence entropies with this procedure we destroy any correlation between any two columns *i *and *j *and therefore obtain distributions for MI_*ij *_with respect to naturally occurring, independent symbol frequencies. From this distribution we can easily compute *Z *values for any MI_*ij *_value by(2)

where  is the average of the MI values for the shuffled columns *i *and *j*, and *σ*(*MI*_*ij*_) the standard deviation of this sample. Note that this has to be done for each column pair (*i*, *j*) independently. This protocol is also implemented in the program micato and it can be loaded into our visualization program as a weighting scheme.

## Results and Discussion

**Figure 1:**
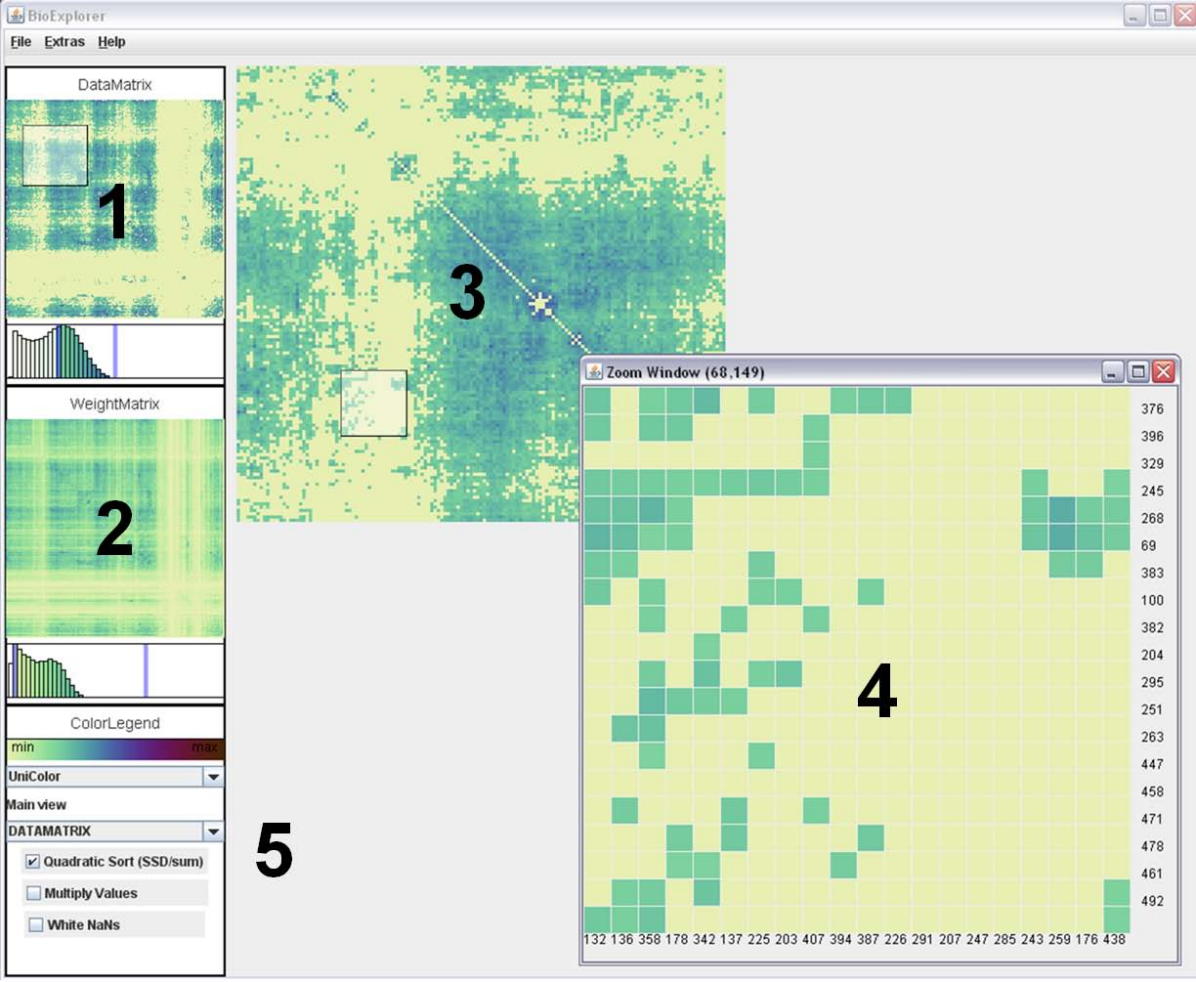
**Matrix Visualization Tool**. This image shows the visualization application we implemented. It basically follows a zoomable user interface (ZUI) approach. (1,2): Overview images of the raw and weight matrix data using color coding. Histograms shown below allow interactive data selection. (5): options for selecting which matrix to show enlarged; selection from choice of color mapping schemes; application of sorting algorithm. Drawing a rectangle in either (1) oder (3) shows the selected matrix area enlarged in (4).

Figure 1 shows a screenshot of the matrix visualization tool we implemented. To support the flexible usage of the system, an intuitive user interface is provided. Two side panels (1,2) visualize a preview of the two matrices. The user can zoom into any of these, with the zoomed areas being continuously updated in the zoom display area (3). Again, from this a region can be selected for further drill-down (4). While both matrices can be browsed individually, it is also possible to combine them in a joint image by multiplication. The display can also be filtered to show only data falling into user-defined intervals. To this end, it is possible to specify the filtering intervals directly in the histograms accompanying the two matrix previews in the left panel. To maintain context, the selected areas in the histograms are highlighted. Finally, an option panel (5) offers to select from a choice of color scales, select or de-select multiplication of the matrices, and optionally, sort the rows and columns of the display. Numeric values are shown in textual form by mouse-over.

The implementation of all components like user interface, data storage and algorithms is kept modular, so it is easy to adopt the system to upcoming needs. This includes e.g., additional matrix ordering methods, color schemes or data filtering mechanisms. Currently, filtering can be done in two steps. Firstly, by setting a maximum and minimum value of the mutual information. Secondly, by doing the same for the *Z*-score. By this the *Z*-score can be used as filter or additionally as a weight for controlling the color saturation of the corresponding matrix entries.

The visualization system can be used additionally with any matrix valued data set beyond co-evolutionary studies. This is achieved by specification of a simple file format for matrix data. The software assumes the raw matrix and weight data to be contained in plain ASCII files, where each row in the file contains one matrix cell entry of the form <*row column value *>.

### Example Protocol

Codoñer et al. [1], as well as other authors [17] have argued that intramolecular co-evolution typically results from a superposition of various biological and biochemical influences, which depend highly on the system under investigation. In fact, the decomposition is the key analysis task in co-evultionary studies. Explorative analysis can then be used for hypothesis creation [18] on the origin of such influences. We applied the software package to a sequence set of variants of the acetylcholinesterase (AChE) (Held S, Hoffgaard F, Hamacher K: Biophysical Annotation of Molecular Coevolution of Acetylcholinesterase, submitted).

**Figure 2:**
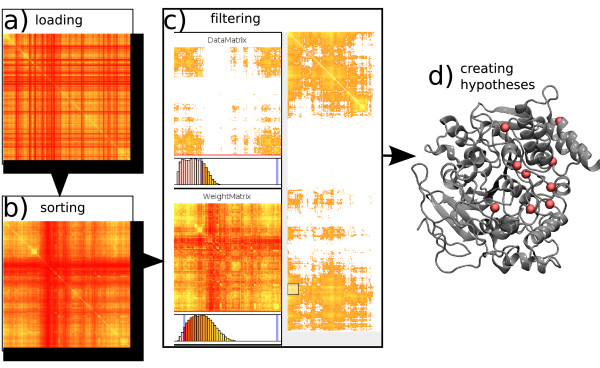
**Matrix Sorting & Filtering for AChE**. A workflow using the software package decomposed into sequential steps a)-d). The sorting and filtering algorithms are the important steps to extract evolutionary signals. (a) shows the mutual information matrix in its generic order; (b) shows the matrix sorted with our quadratic sorting algorithm. Rows and columns are reordered, so that similar ones are next to each other; (c) shows a screenshot of the filtered and sorted data, revealing an interesting portion of residues in the molecule (small box), which are known to form the peripheral anionic site. Filtering was done by setting ranges to be displayed to appropriate values (in the two histograms on the left); (d) the residues found in step (c) to be significantly and highly co-evolving.

Figure 2 illustrates this example application of the system and the steps undertaken to identify an important subset of highly co-evolving residues. Figure 2(a) shows the input mutual information matrix. A sorted version of the matrix is shown in Figure 2(b). The user can detect an interesting cluster of residues in an area that is extracted by filtering for high *Z *scores and high MI values as illustrated in Figure 2(c). We show how the software can be used to restrict the display to the supposedly important ranges of MI and *Z *scores. In part (d) of the same figure we show the residues marked in the molecular structure. These are (for the sequence of *T. californica*) Y70, V236, N280, F284, F288, G335, S345, V360, Q374. Most of these residues belong to the peripheral anionic site (PAS) site of AChE, which is important for establishing contact with the substrate [19]. Also, various AChE inhibitors bind to this site, suggesting a partial explanation why evolutionary signals occur at these spots. The involved residues are subject to a co-evolutionary pressure, the origin of which one can now start to investigate based on the insight we gained from the application of MIMatrixViz to the AChE sequence data.

## Future Trends & Intended Use

The implemented routines can be used in studies on molecular (co-)evolution by working on provided FASTA-files. The MI computation can be run in batch-mode to allow for compute cluster usage. The output of computed mutual information values and their weighting by *Z*-scores can be opened by the interactive matrix visualization software provided. There, as described above the user can navigate the vast amount of data, applying filters in sequence space and in value space, and using *Z*-scores (or other externally provided weights) to estimate and visualize the statistical significance of the mutual information values.

Typically, the visual analytics approach does not guarantee to reveal all relevant features of empirical data. However, in a generic biological application one does not know beforehand what signals to look for. This renders automatic processing ineffective and one has to resort to visual and interactive inspection. Future work includes extending the functionality of the visualization software with additional functionality. First, additional matrix sorting algorithms with user-settable sorting criteria should be included, allowing the user to take suitable views on the data set. The matrix display should be extended by side views showing the similarity of rows and columns as well as the reordering (confusion) index in case the matrix has been sorted. In the long run, the system should be integrated with additional relevant meta data, and linked with additional viewing components such as 3D molecular viewers. Our software is provided as Java Bytecode. The sourcecode can be made available upon request. We are also open for collaboration aiming at improving the functionality of the software and applying it to new use cases.

## Conclusions

In the MIMatrixViz package we provide routines to compute mutual information of evolutionary dynamics in molecules. The package is capable of normalizing those values and therefore accounts for finite-sized data sets. The visualization part is separated from this to allow batch-usage on servers and clusters for sufficient statistics. The visual approach allows to interactively explore the data, and investigate patterns, structures, and particular interesting spots within the mutual information matrices.

The user can generate graphics and filtered data sets with the package in publication ready quality. To this end, the application allows the user to export matrix images in the lossless PNG file format, and to export selected data subsets as plain ASCII files.

Other matrix-oriented data, as e.g., obtained by DNA microarray experiments, can be visually analyzed with the tool, too. External knowledge can be incorporated by the weight matrix to augment the insight one gains from the expression levels detected at the feature sites. Potential scenarios include phylogenetic likelihoods for particular hits on reporters, gauging bias to cope with potential shortcomings in the production and/or binding processes.

## Availability and Requirements

**Project name: **MIMatrixViz

**Project home page: **http://www.gris.informatik.tu-darmstadt.de/projects/vsa/matrixvis/

**Operating system: **Platform independent (Requires a Java Virtual Machine (JVM) on the target system)

**Programming language: **Java

**Requirements: **Java Runtime Environment ≥ 1.6

**License**: GPL for academic users.

**Any restrictions to use by non-academics: **For commercial applications of MIMatrixViz, please contact the authors.

## Authors' contributions

SB, PB, and TS implemented the visualization software. SH tested the software. KH formulated the project and tested the software. All authors wrote the paper. All authors read and approved the final manuscript.
